# Increases in Myocardial Workload Induced by Rapid Atrial Pacing Trigger Alterations in Global Metabolism

**DOI:** 10.1371/journal.pone.0099058

**Published:** 2014-06-16

**Authors:** Aslan T. Turer, Gregory D. Lewis, John F. O'Sullivan, Sammy Elmariah, Jessica L. Mega, Tayo A. Addo, Marc S. Sabatine, James A. de Lemos, Robert E. Gerszten

**Affiliations:** 1 Department of Medicine, Division of Cardiology, University of Texas Southwestern Medical Center, Dallas, Texas, United States of America; 2 Department of Internal Medicine, Division of Cardiology, Massachusetts General Hospital, Boston, Massachusetts, United States of America; 3 Brigham and Women's Hospital, Boston, Massachusetts, United States of America; KRH Robert Koch Klinikum Gehrden, Germany

## Abstract

**Objective:**

To determine whether increases in cardiac work lead to alterations in the plasma metabolome and whether such changes arise from the heart or peripheral organs.

**Background:**

There is growing evidence that the heart influences systemic metabolism through endocrine effects and affecting pathways involved in energy homeostasis.

**Methods:**

Nineteen patients referred for cardiac catheterization were enrolled. Peripheral and selective coronary sinus (CS) blood sampling was performed at serial timepoints following the initiation of pacing, and metabolite profiling was performed by liquid chromatography-mass spectrometry (LC-MS).

**Results:**

Pacing-stress resulted in a 225% increase in the median rate·pressure product from baseline. Increased myocardial work induced significant changes in the peripheral concentration of 43 of 125 metabolites assayed, including large changes in purine [adenosine (+99%, p = 0.006), ADP (+42%, p = 0.01), AMP (+79%, p = 0.004), GDP (+69%, p = 0.003), GMP (+58%, p = 0.01), IMP (+50%, p = 0.03), xanthine (+61%, p = 0.0006)], and several bile acid metabolites. The CS changes in metabolites qualitatively mirrored those in the peripheral blood in both timing and magnitude, suggesting the heart was not the major source of the metabolite release.

**Conclusions:**

Isolated increases in myocardial work can induce changes in the plasma metabolome, but these changes do not appear to be directly cardiac in origin. A number of these dynamic metabolites have known signaling functions. Our study provides additional evidence to a growing body of literature on metabolic ‘cross-talk’ between the heart and other organs.

## Introduction

There has been a long history of investigation into myocardial substrate selection and energetics during periods of increased cardiac work and in the context of myocardial ischemia. In recent years, global metabolite profiling has been performed to gain insights into additional metabolic pathways active during periods of myocardial stress, e.g. ischemia [1]–[3], infarction [4], and exercise [5]. These studies have demonstrated that the circulating metabolites change in response to such perturbations.

To what degree changes in the peripheral metabolome under these conditions reflect changes in cardiac metabolism versus peripheral metabolism is not clear. Furthermore, it is unknown whether changes in peripheral metabolism can occur directly as a consequence of increases in myocardial work. This is an important question, as it is becoming increasingly recognized that the heart is able to influence metabolic events in non-cardiac tissues by endocrine effects. Currently, the best characterized of these pathways is the influence of the heart on adipose tissue via natriuretic peptides, resulting in lipolysis and release of free fatty acids [6]. However, additional humoral pathways linking both skeletal muscle [7] and the heart [8] to energy homeostasis have recently been described. Taken together, these lines of evidence suggest that increases in cardiac work alone may be able to induce metabolic effects on peripheral tissues in humans. However, disentangling such changes in the metabolome is challenging. In most instances such as during exercise, increasing cardiac work involves increasing work of other tissues, such as peripheral muscle. Further, identifying the origin of metabolite changes- i.e. from the heart or peripheral tissues- requires collection of cardiac-specific effluent.

Using a novel human pacing model coupled with a mass spectrometry-based metabolite profiling platform, we tested whether the modulation of cardiac work (with or without the development of cardiac ischemia) results in detectable changes in the plasma metabolome. Further, by using coronary sinus catheterization we compared changes in the plasma levels of metabolites in cardiac-specific effluent with peripheral arterial samples to explore the dynamic coupling between myocardial work and systemic energetics.

## Methods

### Pacing Protocol Patient Population

Nineteen patients referred for coronary angiography for evaluation of stable angina at Parkland Memorial Hospital were enrolled. Patients with valvular heart disease, atrial fibrillation, prior CABG, a history of heart failure, acute coronary syndrome, or baseline left bundle branch block were excluded. The protocol was approved by the University of Texas Southwestern Institutional Review Board, and all subjects provided written informed consent.

### Study protocol

**Figure 1:**
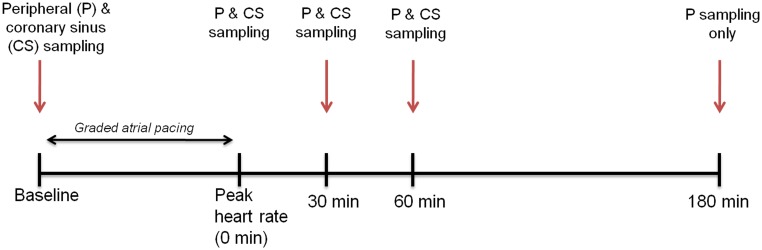
Study schema and timing of sample acquisition.

The details of the protocol have been published previously [9] and are summarized in **Figure 1**. All patients had beta-blockers and nitrates held for at least 24 hrs prior to catheterization, and participants were brought to the cardiac catheterization suite in an overnight-fasted state. A 6Fr arterial cannula was placed in either the brachial or femoral artery while a 7Fr Zucker catheter (allowing for simultaneous blood sampling and pacing) was placed in the coronary sinus (CS) from a brachial vein. Following the acquisition of simultaneous baseline blood samples from both the peripheral artery and CS, the pacing protocol was initiated. The atrium was paced at 20 beats per minute (bpm) above the resting heart rate, and the rate was increased by 20 bpm every three minutes until the patient developed either angina-like chest pain or reached a target heart rate of 160 bpm. Atropine (0.5–1 mg) was administered to six participants who developed atrioventricular block before reaching target heart rate. At peak heart rate, a matched set of arterial and CS blood samples was again obtained. Additional paired arterial and CS samples were taken 30- and 60-minutes after the cessation of pacing. The CS catheter was then removed and coronary angiography was then performed to define coronary anatomy. Significant coronary disease was defined as ≥75% luminal diameter narrowing of at least one major epicardial coronary artery by angiogram. Additional samples of peripheral blood were then obtained at 180 minutes following cessation of pacing. No coronary interventions (i.e. angioplasty or stenting) were performed on the day of the study.

### Control population

To control for the effects of the catheterization itself, a set of historical control samples from patients (n = 9–25 per analyte) undergoing diagnostic catheterization at Massachusetts General Hospital was analyzed. Samples were obtained at the onset of the procedure and 60-minutes later. All patients presented without evidence of acute coronary ischemia. The clinical characteristics and collection methodology for these control populations has previously been reported [4], [10].

### Sample preparation

Blood samples were collected into EDTA tubes and placed in an iced saline bath until processing occurred (<1 hr after collection). Samples were centrifuged and plasma was stored at −70°C until assays were performed. Plasma samples underwent a single thaw cycle for all measurements.

### Metabolite Analysis

Metabolite profiling was performed by liquid chromatography-mass spectrometry (LC-MS) methods as previously described [4] and also summarized in **Text S1 in File S1**. Peripheral and CS measurements were expressed as percent changes over time by normalizing to the baseline value for each timepoint. AV differences, expressed as percentages of extraction or release, were generated by taking the difference of the [metabolite]^peripheral^– [metabolite]^CS^ and dividing by the [metabolite]^peripheral^. Thus, extractions are denoted by a positive percentage, while elutions are negative.

### Assessment of myocardial ischemia

Patients were categorized into two groups based on the development of cardiac ischemia during cardiac pacing, using a combination of metabolic, anatomic and biomarker criteria [9]. Under normal aerobic conditions, the heart is a net consumer of lactate while areas of ischemic myocardium elute lactate from the anaerobic metabolism of pyruvate. Five of the 19 patients had no significant CAD and no net lactate elution after pacing, strongly suggesting the absence of cardiac ischemia. Seven of the 19 patients had significant CAD and a conversion to anaerobic metabolism (net lactate release) indicating clear myocardial ischemia. Of the remaining seven patients all had significant CAD, but no net lactate release, suggesting either absence of ischemia or an area of ischemia that was too small to result in a net lactate release detectable in the CS given uptake in the remainder of the non-ischemic myocardium. To distinguish between these two alternatives, we further classified patients based on whether they had pacing-induced elevations in cardiac troponin T concentrations over the upper-limit of normal (>13 pg/mL) detectable by a highly sensitive assay (Elecsys-2010 Troponin T hs STAT Roche Diagnostics), suggestive of ischemia in the absence of frank myocardial necrosis, as previously described in this patient cohort [9]. Using these criteria, ten patients were classified as having developed myocardial ischemia and nine did not.

### Statistical analysis

Clinical and procedural characteristics are presented as median (25^th^, 75^th^ percentile) or percentages, as appropriate. Since metabolite values were non-normally distributed, we also report values as median and utilized non-parametric statistical methods for comparisons. The Wilcoxon signed-rank test was used to test for significant transcoronary gradients (i.e. significant myocardial uptake or elution) by comparing the observed AV difference with the null hypothesis (gradient of 0). Between-group comparisons of patients who did and did not meet criteria for ischemia were performed using the Mann-Whitney U-test. Relative changes in metabolites over time were assessed by Friedman's Chi-Square test. To control for the false discovery rate (FDR), a Benjamini-Hochberg correction was performed and the adjusted p-values (i.e. the q-values) are reported. Although statistical significance was defined as a corrected p-value of <0.05, only metabolites with absolute changes >10% were considered significant even if the p value was <0.05. The heat map was constructed with R using RStudio and RColorBrewer. All other statistical analyses were performed using SAS 9.2.

## Results

### Study population and pacing results

The baseline characteristics of the study population are shown in **Table 1**. Hypertension (74%), hyperlipidemia (68%), smoking (58%) and diabetes mellitus (42%) were common among participants. Fourteen of the 19 patients (74%) had at least one significant coronary stenosis (≥75%) on angiography.

**Table 1:** Demographic and clinical characteristics of the study population.

Clinical Characteristic	Entire Cohort (n = 19)	Ischemia Grouping	
		Non-Ischemic (n = 10)	Ischemic (n = 9)
Age (years)	51 (47,56)	49 (47,52)	56 (51,57)
Gender (no., % female)	7 (37)	4 (40)	3 (33)
Race/ethnicity			
White	6 (32)	4 (40)	2 (22)
Black	8 (42)	4 (40)	4 (44)
Hispanic	5 (26)	2 (20)	3 (33)
Hypertension (%)	14 (74)	8 (80)	6 (67)
Hyperlipidemia (%)	13 (68)	6 (60)	7 (78)
Diabetes mellitus (%)	8 (42)	3 (30)	5 (56)
Tobacco use (%)	11 (58)	7 (70)	4 (44)
Angiography			
No. diseased vessels			
0	5 (26)	5 (50)	0 (0)
1	9 (53)	2 (20)	7 (78)
2	4 (16)	3 (30)	1 (11)
3	1 (5)	0 (0)	1 (11)
Diseased vessel			
Left Anterior Descending	8 (57)	1 (10)	7 (78)
Left Circumflex	5 (36)	3 (30)	2 (22)
Right Coronary Artery	6 (43)	3 (30)	3 (33)
Pacing-response			
Baseline heart rate (bpm)	68 (58,80)	68 (62,76)	69 (58,80)
Rate·pressure product at baseline (bpm·mmHg)	10240 (8690,11704)	11220 (8960,11988)	10078 (8690,11480)
Peak pacing heart rate (bpm)	143 (132,160)	141 (137,160)	138 (126,154)
Rate·pressure product with pacing (bpm·mmHg)	22475 (19454,25584)	21528 (19454,25920)	22820 (20128,24640)
Chest pain	13 (68)	6 (60)	7 (77)
ST-segment depression	9 (47)	5 (50)	4 (44)
Lactate elution	7 (37)	0 (0)	7 (78)
Peak hs-cTnT>upper limit of normal (>13 pg/mL)	7 (37)	0 (0)	7 (78)

Prior to the onset of pacing, the median heart rate and blood pressure were 68 bpm and 150/78 mmHg. This increased to 143 bpm and 155/100 mmHg at peak pacing, representing a 225% increase in the median rate·pressure product. Nine of the patients (47%) were classified as having developed cardiac ischemia at peak pacing by either lactate elution or hs-cTnT criteria. The other ten patients (53%) did not develop overt ischemia; five of these patients had no significant CAD. The hemodynamic and clinical variables are summarized in **Table 1**.

### Changes in the Peripheral Metabolome Associated with Pacing-Stress

**Figure 2:**
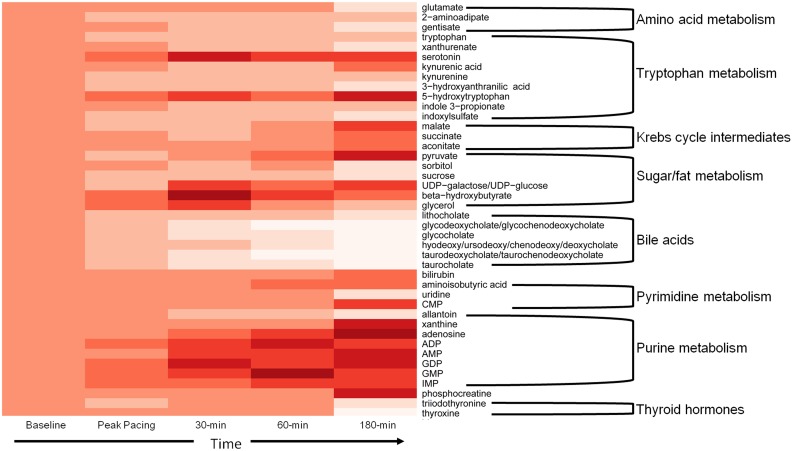
Heat map describing significant changes in peripheral concentrations of metabolites over the course of the experiment. Only metabolites with absolute changes >20% are shown. Darker red indicates increases, while lighter red indicates decreases in concentrations.

Increased myocardial work induced significant changes in the peripheral concentration of 43 of 125 metabolites assayed (**Table 2** and **Table S1 in File S1**). Among the metabolites displaying significant changes were a number of amino acids, tryptophan hydrolase metabolites (e.g. serotonin, 5-hydroxytryptophan), glycolysis/carbohydrate metabolites and thyroid hormones. The most prominent changes were seen with the bile acids, which uniformly fell between 30–63% by 180-minutes, and the purine metabolites [adenosine (+99%, p = 0.006), ADP (+42%, p = 0.01), AMP (+79%, p = 0.004), GDP (+69%, p = 0.003), GMP (+58%, p = 0.01), IMP (+50%, p = 0.03), xanthine (+61%, p = 0.0006)], which rose significantly. The changes in the peripheral metabolites over time are summarized in **Figure 2**. As a point of reference, only three of the total number of analytes which changed in the pacing cohort demonstrated directionally similar changes in the control cohort at 60-minutes (**Table S2 in File S1**)

**Table 2:** Significant metabolite changes from baseline measured in peripheral blood.

	Δ% peak pacing	Δ% 30-minutes	Δ% 60-minutes	Δ% 180-minutes	p-value
**Amino Acid Metabolism**					
2-aminoadipate	−8%	−3%	−10%	−21%	0.03
α-hydroxybutyrate	2%	9%	10%	8%	0.03
gentisate	0%	−14%	−17%	−34%	0.0006
glutamate	0%	10%	3%	−35%	0.001
**Tryptophan Hydrolase Metabolites**					
3-hydroxyanthranilic acid	−7%	−8%	−9%	−29%	0.0006
5-hydroxytryptophan	21%	41%	34%	78%	0.02
indole 3-propionate	10%	−12%	−12%	−20%	0.007
indoxylsulfate	−7%	−12%	−8%	−29%	0.04
kynurenic acid	1%	−2%	−7%	19%	0.03
serotonin	37%	63%	57%	57%	0.002
xanthurenate	4%	−11%	−12%	−29%	0.003
**Purine Metabolism**					
adenosine	2%	23%	54%	99%	0.006
ADP	30%	55%	65%	42%	0.01
allantoin	2%	−7%	−10%	−26%	0.006
AMP	12%	53%	40%	79%	0.004
GDP	22%	63%	55%	69%	0.003
GMP	21%	59%	92%	58%	0.01
IMP	24%	32%	44%	50%	0.03
inosine	8%	−18%	−6%	7%	0.04
xanthine	6%	10%	17%	61%	0.0006
**Pyrimidine Metabolism**					
aminoisobutyric acid	6%	11%	21%	32%	0.0006
uridine	3%	3%	3%	−40%	0.0006
**Glycolysis/Carbohydrate Metabolism**					
fructose/glucose/galactose	0%	1%	0%	−10%	0.03
lactate	0%	−8%	−11%	7%	0.002
pyruvate	−5%	1%	32%	69%	0.002
sorbitol	−1%	−4%	8%	−25%	0.0006
sucrose	−11%	−17%	−18%	−39%	0.01
UDP-galactose/UDP-glucose	−6%	49%	19%	58%	0.03
**Bile Acid Metabolism**					
glycocholate	−6%	−25%	−30%	−51%	0.009
glycodeoxycholate/glycochenodeoxycholate	−11%	−40%	−58%	−54%	0.002
hyodeoxy/ursodeoxy/chenodeoxy/deoxycholate	−6%	−14%	−26%	−48%	0.0006
lithocholate	−8%	−3%	−16%	−30%	0.01
taurine	3%	12%	13%	8%	0.007
taurocholate	−3%	−39%	−29%	−54%	0.02
taurodeoxycholate/taurochenodeoxycholate	−9%	−40%	−58%	−63%	0.009
**Lipolysis**					
β-hydroxybutyrate	26%	87%	48%	38%	0.0006
glycerol	35%	48%	16%	−5%	0.006
**Thyroid Hormones**					
thyroxine	2%	0%	−1%	−60%	0.002
triiodothyronine	−6%	−1%	−1%	−39%	0.005
**Other Metabolic Pathways**					
aconitate	−1%	2%	0%	20%	0.04
α-glycerophosphocholine	2%	6%	13%	−7%	0.02
phosphocreatine	−1%	9%	7%	62%	0.002
thiamine	−1%	0%	7%	−17%	0.04

Median percent changes are shown. P-values refer to differences over time by Friedman's test and are adjusted for false discovery rate. Results from all the analytes which were assayed are displayed in **Table S1**.

**Figure 3:**
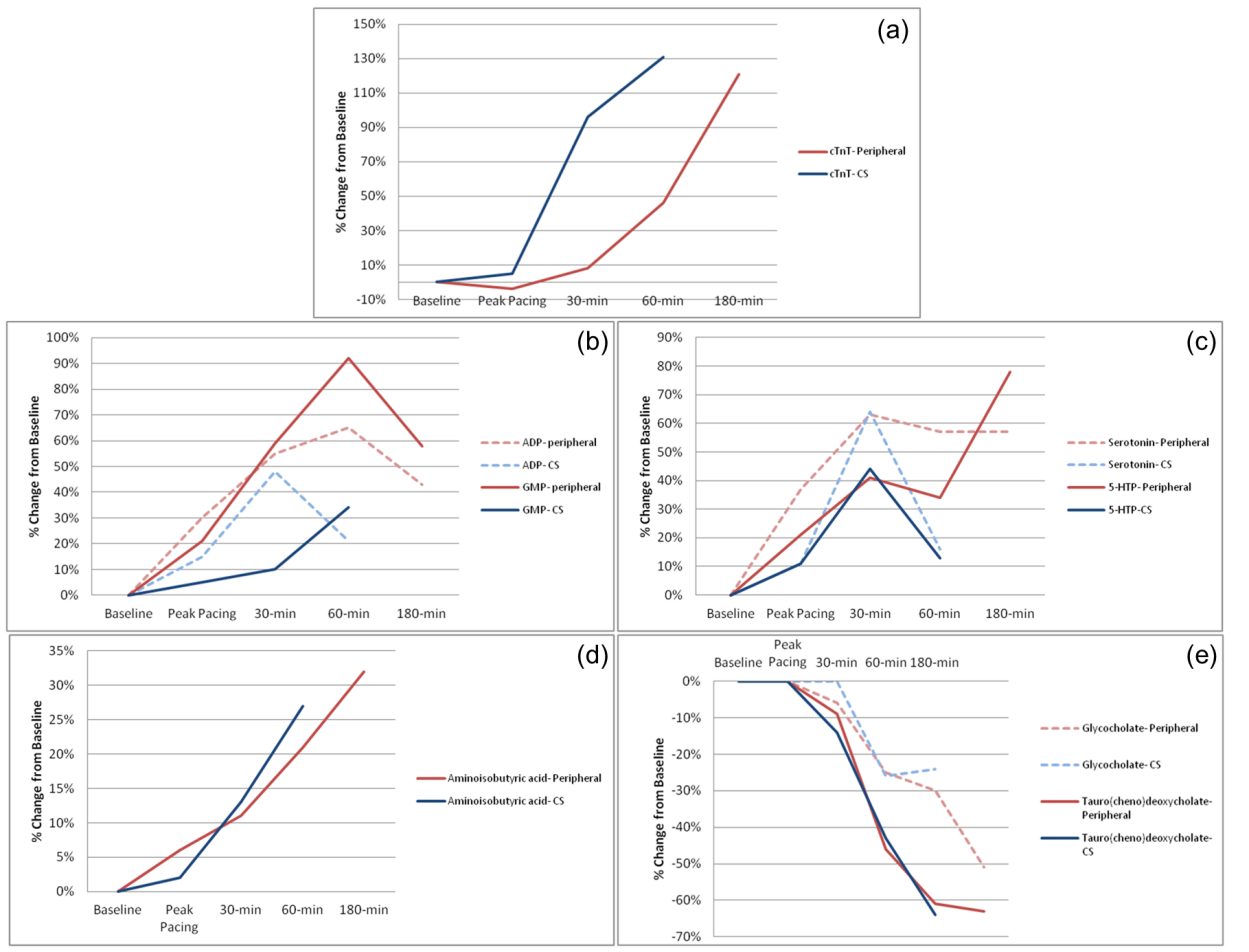
Median relative changes in the peripheral and CS concentrations of (a) cardiac troponin T, as measured by a highly-sensitive assay. Levels of this cardiac-specific biomarker are increased earlier in the CS, but are eventually mirrored in the peripheral blood. This is in distinction to the observed changes in e.g. (b) purine metabolites, (c) tryptophan hydrolase metabolites, (d) aminoisobutyric acid, or (e) the bile acids.

In order to ascertain whether the changes observed in the peripheral blood metabolome were due to excess release from the heart during or following pacing, we quantified metabolites from CS effluent and compared the changes with those observed in the periphery. In distinction to the cardiac-specific biomarker, cardiac troponin T, the CS changes in these metabolites qualitatively mirrored those seen in the peripheral blood in both timing and magnitude (**Figure 3**, **Table S1 in File S1**), suggesting the heart was not the major source of the metabolite release.

### Metabolite Changes in Response to Myocardial Ischemia

**Figure 4:**
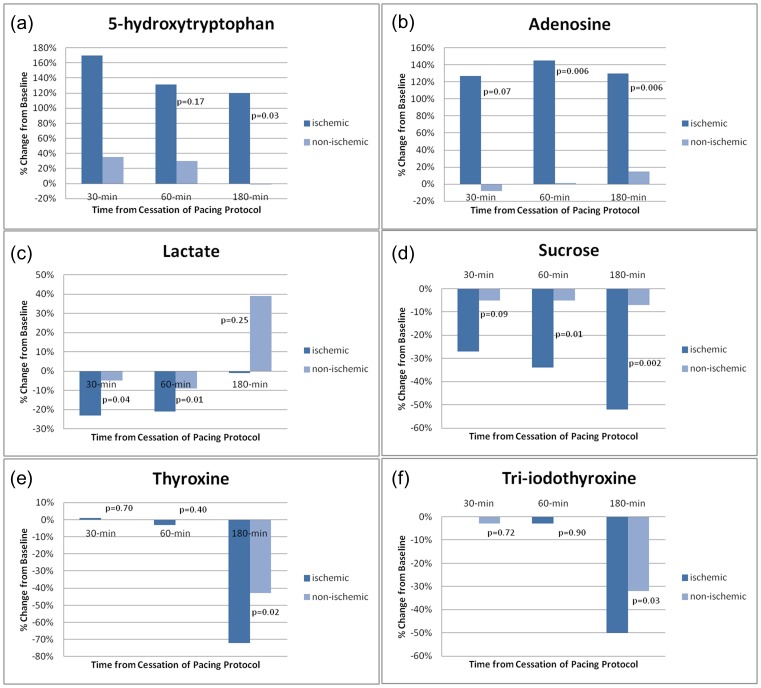
Metabolites (a–f) which displayed differential changes in peripheral concentrations after rapid pacing, stratified by with the presence (n = 9) or absence (n = 10) pacing-induced myocardial ischemia. Median changes from baseline are shown for each timepoint.

To determine whether the changes in the peripheral metabolic signature were due to increased myocardial work or overt cardiac ischemia, we stratified the cohort based on the presence of pacing-induced ischemia. Of the 43 metabolites measured from peripheral blood which were noted to have changed significantly over the course of study, only six were significantly different between ischemic and non-ischemic patients following termination of the pacing protocol (**Figure 4**).

Large differences in peripheral concentrations of adenosine (ischemic +130% vs non-ischemic +15% at 180-minutes, p = 0.006) and 5-hydroxytryptophan concentrations (ischemic +120% vs non-ischemic −1% at 180-minutes, p = 0.03) were observed. However, there was no significant difference beween ischemic and nonischemic subjects in transcoronary elution of either 5-hydroxytryptophan (e.g. −16% vs −1% at 60-minutes, p = 0.92) or adenosine (e.g. −41% vs −21% at 60-minutes, p = 0.92) at any timepoint after pacing implying a non-cardiac source for this differential response.

Although not reflected by differences in peripheral concentrations, the cardiac elution/uptake of several analytes differed based on the presence or absence of ischemia. Most of these changes were only observed at peak pacing (**Table 3**). In addition to the expected higher release of lactate, we documented greater release of alanine (−11% vs −3%, p = 0.04), α-ketoglutarate (−17% vs −1%, p = 0.03), fumarate (−22% vs 14%, p = 0.0003), and lower uptake of glutamate (12% vs 38%, p = 0.03) among those with ischemia compared to those without; there was also a trend towards higher succinate release with ischemia (−142% vs-38%, p = 0.05). Taken together, these findings demonstrate a greater loss of Krebs' cycle intermediates with ischemia.

**Table 3:** Metabolites in which the transcoronary gradient was significantly different at peak pacing between patients with or without demonstrable coronary ischemia.

Analyte	Baseline			Peak Pacing		
	Ischemic	Non-ischemic	p-value	Ischemic	Non-ischemic	p-value
glycine	−3%	0%	1.00	−7%	2%	**0.03**
alanine	−7%	−2%	0.24	−11%	−3%	**0.04**
serine	−1%	1%	0.51	−9%	5%	**0.04**
threonine	1%	2%	0.68	−9%	−1%	**0.02**
glutamate	32%	45%	0.57	12%	38%	**0.03**
lysine	0%	1%	0.45	−11%	4%	**0.02**
phenylalanine	2%	0%	0.55	−8%	1%	**0.04**
aminoisobutyric acid	−3%	6%	0.32	−17%	4%	**0.003**
kynurenine	−1%	−1%	0.78	−9%	−1%	**0.04**
lactate	24%	23%	0.28	−16%	5%	**0.04**
α-KG	4%	−16%	**0.04**	−17%	−1%	**0.03**
fumarate	12%	13%	0.60	−22%	14%	**0.0003**

Median AV extraction (positive percentage) or release (negative percentage).

## Discussion

In this study we observed that increases in cardiac work from rapid pacing resulted in a significant and prolonged change in the metabolic signature of the peripheral circulation. We documented increases in a number of metabolites broadly involved in energetics (carbohydrate metabolism, nucleoside phosphates) and suppression of circulating bile acids and thyroid hormones. Somewhat surprisingly, despite the specific nature of the stress (i.e. targeting the heart) the myocardium did not appear to be the major source of these metabolite changes. Furthermore, although a few of the metabolite changes differed among patients who developed cardiac ischemia with stress, the majority were not different, suggesting the major cue for these changes was the increase in cardiac work and not myocardial ischemia, per se.

Metabolic profiling of plasma after stress testing [1] and marathon-running [5] have previously demonstrated significant changes accompanying exercise in purine metabolites and Krebs' cycle intermediates. It remained unclear to what degree these changes reflected alterations in cardiac- versus peripheral (e.g. skeletal muscle) metabolism. In the present experiment, which increased cardiac work without increasing work of other organs, directionally similar trends were noted among a number of the same metabolite classes. However, since similar magnitudes of change were observed in peripheral and cardiac-specific effluent, and since the cardiac changes did not precede the peripheral changes, we conclude that the heart was not the major source of the changes in the peripheral metabolome. This suggests that increases in myocardial work can induce pleiotropic effects on distant tissues.

Appreciation of the ability of the endocrine heart to influence peripheral metabolism is widening. The natriuretic peptides cause lipolysis and browning of white adipose tissue [6]. They serve as a well-characterized example of how changes in cardiac work (reflected in wall stress) may influence local and systemic metabolic events through the influence of humoral signaling pathways. Similarly, the skeletal muscle-derived protein, irisin, has been proposed as a link between exercise and alterations in adipose tissue metabolic phenotype [7]. Most recently, *cardiac-specific* expression of MED13, a subunit of the Mediator complex which modulates thyroid hormone-dependent transcription, was shown to influence *systemic* metabolism [8]. Specifically, myocardial overexpression of MED13 (or inhibition of its regulatory microRNA, miR-208a) led to resistance to obesity and systemic insulin-resistance when challenged with high-fat feeding. The opposite finding, i.e. susceptibility to obesity, was observed in the condition of myocardial MED13-deletion. These results are broadly in line with the observations made here- namely that peripheral metabolism is coupled with myocardial function. It remains unclear in this context, however, whether these pacing-induced effects are from cardiac-release of a humoral substance(s) or other factors such as sympathetic outflow. Alternatively, the trigger for these changes could be from cardiac-vascular interplay, such large vessel distention, differential shunting of blood or sudden increases in cardiac output from increases in heart rate.

Although many changes can be observed in the levels of peripheral metabolites, it is not known what, if any, function they may be serving in plasma. Some metabolite changes may simply represent byproducts of tissue metabolic processes which are subsequently cleared. The fact that most of the major changes occur after several hours would argue against this possibility. Several metabolites we observed to have changed have been ascribed signaling roles. Both the thyroid hormones, as well as the bile acids [11], [12], which were observed to have dropped significantly following pacing, have important roles as hormones involved with substrate utilization and energy homeostasis. To this point, it is also notable that the levels of a number of high-energy phosphate-coupled nucleosides gradually increase after pacing. The fact that levels of these metabolites start changing early but do not peak for several hours after the cessation of pacing suggests that the effect is not simply due to a brief period of increased cardiac output. Adenosine, levels of which doubled over the period of observation, also has well-described signaling functions regulating both vascular tone and pre-conditioning. However, unlike most other metabolites measured, adenosine release may have been preferentially observed in those with coronary ischemia, a finding consistent with prior reports [13], [14].

Several limitations, mainly related to the logistic complexities of the study design, must be acknowledged. First, the CS catheters were only in place for the first 60 minutes of the study. We are, therefore, unable to report on cardiac metabolite release which may have taken place beyond the first hour which may have contributed to the peripheral signal observed hours later. Second, our patients did receive exogenous medications, such as mild sedatives, aspirin and heparin, with the procedure. Heparin is known to briefly promote lipolysis through release of lipoprotein lipase and increase circulating free fatty acid levels [15]. For this reason we did not perform lipidomic measures, beyond glycerol and ketone measurements. Additionally, despite the inclusion of a limited control group which did not receive the pacing protocol, we cannot exclude some effects from the catheterization itself or the requirement of fasting prior to the procedure in some of the findings (e.g. for bile acids). Next, unambiguously defining coronary ischemia is difficult based on any one or several clinical or biochemical parameters. Chest pain and EKG changes are non-specific [16]. Net coronary sinus lactate elution is relatively specific but cardiac glycolysis can be effected by co-morbid conditions, such as diabetes [17], and detection of net lactate efflux may not be sensitive to small areas of myocardial ischemia. We, therefore, supplemented the definition with high-sensitivity. Finally, our study was exploratory, and the sample size was small as a result of the complex design. As a result, the findings should be considered hypothesis generating. The small sample size limits our statistical power to detect significant changes in analytes with smaller relative changes and our ability to detect differences between those patients with and without pacing-induced ischemia. Furthermore, our study participants generally had both clinical indication for cardiac catheterization and risk factors for atherosclerosis and whether and to what degree such metabolic perturbations could be seen in a healthy population are not known.

In conclusion, we have demonstrated that isolated increases in the myocardial rate·pressure product can induce dramatic changes in the plasma metabolome. Interestingly, most of these changes do not appear to be due to cardiac release, but rather are caused by changes in metabolism in peripheral tissues in response to increases in myocardial work. A number of these changes mirror those previously observed during exercise. Several of the metabolites observed to have changed significantly have known signaling functions. Our study provides additional evidence to support metabolic ‘cross-talk’ between the heart and other tissues.

## Supporting Information

File S1 **Supplemental Methods and Tables.**
(DOCX)
