# Supplementary material for: Increases in Myocardial Workload Induced by Rapid Atrial Pacing Trigger Alterations in Global Metabolism
Source: PLoS One. 2014 Jun 16;9(6):e99058. doi: 10.1371/journal.pone.0099058 (PMC4059652; doi:10.1371/journal.pone.0099058)
Supplement: File S1 — Supplemental Methods and Tables. (DOCX) [file pone.0099058.s001.docx]

Supplemental Materials

**Text S1**

**Supplemental Methods**

Metabolite profiling

Blood samples derived from the coronary sinus or peripheral arterial sheath were collected, centrifuged and the plasma stored at -80°C until assayed. Plasma samples were prepared for LC-MS analyses via protein precipitation with the addition of nine volumes of 74.9:24.9:0.2 vol/vol/vol acetonitrile/methanol/formic acid containing two additional stable isotope-labeled internal standards for L-valine-d8 and L-phenylalanine-d8. Formic acid, ammonium acetate, LC-MS–grade solvents and L-valine-d8 were purchased from Sigma-Aldrich. L-phenylalanine-d8 was purchased from Cambridge Isotope Laboratories.

Liquid chromatography-tandem mass spectrometry (LC-MS) data were acquired using a 4000 QTRAP triple quadrupole mass spectrometer (Applied Biosystems/Sciex) that was coupled to a multiplexed LC system comprised of two 1200 Series pumps (Agilent Technologies) and an HTS PAL autosampler (Leap Technologies) equipped with two injection ports and a column selection valve, as previously reported (1). The two pumps were similarly configured for hydrophillic interaction chromatography (HILIC) using 150 x 2.1 mm Atlantis HILIC columns (Waters) and with the same mobile phases (mobile phase A: 10 mM ammonium formate and 0.1% formic acid, v/v; mobile phase B: acetonitrile with 0.1% formic acid, v/v). Multiplexing was used to enable the measurement of 125 metabolite transitions divided between the 2 LC systems, and each sample was injected once on each. The samples were injected directly onto a 150 x 2.0 mm Luna NH2 column (Phenomenex) that was eluted at a flow rate of 400 µL/min with initial conditions of 10% mobile phase A ((20 mM ammonium acetate and 20 mM ammonium hydroxide (Sigma-Aldrich, St. Louis MO) in water (VWR)) and 90% mobile phase B ((10 mM ammonium hydroxide in 75:25 v/v acetonitrile/methanol (VWR)) followed by a 10 min linear gradient to 100% mobile phase A. MS analyses were carried out using electrospray ionization (ESI) and multiple reaction monitoring (MRM) scans in the positive ion mode. Declustering potentials and collision energies were optimized for each metabolite by infusion of reference standards prior to sample analyses. The dwell time for each transition was 30 ms, the ion spray voltage was 4.5 kV, and the source temperature was 425ºC. Internal standard peak areas were monitored for quality control and individual samples with peak areas differing from the group mean by more than two standard deviations were reanalyzed.

The samples were centrifuged (10 min, 15,000*g*, 4 °C), and the supernatants were injected directly. Metabolite concentrations were determined using the standard addition method.

Amino acids, amino acid derivatives, urea cycle intermediates, nucleotides and other positively charged polar metabolites were profiled using 10 μl of plasma.

For organic acids, sugars, bile acids, and other negatively charged polar metabolites, 200 μL of plasma was extracted following a modified Bligh-Dyer method (2), and the resulting aqueous phase was dried down and reconstituted in methanol containing labeled isotope standards (L-Phenylalanine-d_8_ and L-Valine-d_8_).

MultiQuant software (Version 1.1; Applied Biosystems/Sciex) was used for automated

peak integration and metabolite peaks were manually reviewed for quality of integration and compared against a known standard to confirm identity.

For all isotope measurements using these LC/MS methods, peak areas were greater than two orders of magnitude above the lower limit of quantitation (as defined as a discrete peak 10-fold greater than noise) and fell well within the linear range of the dose-response relationship. Data were normalized relative to pooled plasma reference samples that were analyzed in the sample queue after sets of 20 study samples.

References

1. Wang, T.J., Larson, M.G., Vasan, R.S., Cheng, S., Rhee, E.P., McCabe, E., Lewis, G.D., Fox, C.S., Jacques, P.F., Fernandez, C., et al. 2011. Metabolite profiles and the risk of developing diabetes. *Nat Med* 17:448-453.
2. Wahren, J., Felig, P., Cerasi, E., and Luft, R. 1972. Splanchnic and peripheral glucose and amino acid metabolism in diabetes mellitus. *J Clin Invest* 51:1870-1878.

Table S1: Median % (25^th^, 75^th^ quartile) of peripheral and CS changes from baseline in all metabolites measured. *adjusted p-values (i.e. q-values), controlling for False-Discovery Rate

| Metabolite | Site | peak pacing | 30-min | 60-min | 180-min | p-value* |
| --- | --- | --- | --- | --- | --- | --- |
| 1-methylhistamine | Peripheral | -3 (-6,15) | 14 (-12,30) | 7 (-19,35) | 15 (-8,29) | 0.65 |
|  | CS | 1 (-15,3) | 0 (-17,9) | -1 (-27,38) |  |  |
| 2-aminoadipate | Peripheral | -8 (-21.8,3.3) | 3 (-11,10) | -10 (-23,7) | -21 (-31,-7) | 0.03 |
|  | CS | -6 (-17,4) | -6 (-10,3) | 1.8 (-16,15) |  |  |
| 2'-deoxyadenosine | Peripheral | -8 (-23,11) | -11 (-29,7) | -9 (-25,5) | -24 (-44,4) | 0.36 |
|  | CS | -11 (-52,32) | -24 (-45,15) | -8.5 (-36,20) |  |  |
| 2-hydroxyglutarate | Peripheral | -2 (-33,7) | -3 (-26,34) | 9 (-16,28) | -3 (-21,56) | 0.37 |
|  | CS | -6(-13,14) | 2 (-8,24) | 10 (-7,23) |  |  |
| 3-hydroxyanthranilic acid | Peripheral | -7 (-12,1) | -8 (-19,0) | -9 (-23,9) | -29 (-36,-16) | 0.0006 |
|  | CS | -1 (-13,7) | -3 (-11,3) | -6 (-20,6) |  |  |
| 3-hydroxykynurenate | Peripheral | 3 (-27,16) | -7 (-38,23) | -18 (-38,8) | -29 (-54,-4) | 0.70 |
|  | CS | -2 (-27,12) | -8 (-31,25) | -4 (-20,26) |  |  |
| 3-methyladipate/pimelate | Peripheral | -5 (-19,3) | 4 (-13,28) | 4 (-7,35) | 0 (-21,33) | 0.50 |
|  | CS | -5 (-23,10) | -8 (-13,7) | 5 (-18,16) |  |  |
| 4-pyridoxate | Peripheral | 1 (-5,6) | 2 (-8,14) | -3 (-14,6) | -8 (-19,10) | 0.31 |
|  | CS | -2 (-9,5) | -3 (-18,3) | -2 (-17,7) |  |  |
| 5-HIAA | Peripheral | -4 (-8,12) | 7 (-5,14) | -2 (-12,27) | 8 (-10,15) | 0.33 |
|  | CS | 3 (-9,17) | 4 (0,35) | 16 (-1,38) |  |  |
| 5-hydroxytryptophan | Peripheral | 21 (-26,51) | 41 (11,87) | 34 (-7,383) | 78 (-9,127) | 0.02 |
|  | CS | 11 (-24,39) | 44 (-2,175) | 13 (1,189) |  |  |
| 5-methyl-THF | Peripheral | 6 (-27,29) | 25 (0,37) | 22 (-14,52) | 0 (-55,48) | 0.33 |
|  | CS | 12 (-23,58) | 25 (-14,36) | 12 (-12,59) |  |  |
| acetylcholine | Peripheral | -5 (-26,23) | 7 (-24,26) | -6 (-20,7) | 12 (-6,26) | 0.33 |
|  | CS | -4 (-21,27) | 18 (-14,34) | 4 (-5,24) |  |  |
| aconitate | Peripheral | -1 (-7,6) | 2 (-5,9) | -1 (-6,11) | 20 (-5,44) | 0.04 |
|  | CS | -3 (-7,4) | 1 (-8,13) | -2 (-3,10) |  |  |
| adenosine | Peripheral | 2 (-21,41) | 23 (-24,139) | 54 (-7,146) | 99 (15,179) | 0.006 |
|  | CS | 14 (-39,54) | 5 (-27,43) | 35 (-10,92) |  |  |
| adipate | Peripheral | -3 (-14,4) | 3 (-7,19) | 3 (-7,27) | 3 (-18,22) | 0.46 |
|  | CS | -1 (-12,10) | -3 (-9,3) | -3 (-6,12) |  |  |
| ADMA | Peripheral | 4 (-5,11) | 1 (-11,14) | 7 (0,17) | 5 (-5,22) | 0.27 |
|  | CS | -8 (-14,3) | 1 (-10,17) | -4 (-7,7) |  |  |
| ADP | Peripheral | 30 (-34,56) | 55 (19,161) | 65 (0,270) | 43 (7,126) | 0.01 |
|  | CS | 15 (-30,53) | 48 (-27,92) | 21 (-9,130) |  |  |
| alanine | Peripheral | -3 (-8,2) | -7 (-12,1) | -7 (-16,-1) | 0 (-7,25) | 0.04 |
|  | CS | -7 (-12,-2) | -5 (-9,-4) | -5 (-14,-3) |  |  |
| allantoin | Peripheral | 2 (-3,11) | -7 (-15,4) | -10 (-25,7) | -26 (-32,0) | 0.006 |
|  | CS | 3 (-7,23) | -15 (-29,0) | -13 (-23,-3) |  |  |
| alpha-glycerophosphate | Peripheral | -2 (-8,8) | -9 (-15,-2) | -6 (-13,1) | 2 (-9,25) | 0.04 |
|  | CS | -3 (-7,3) | -8 (-22,5) | -6 (-16,12) |  |  |
| alpha-glycerophosphocholine | Peripheral | 2 (-7,14) | 6 (0,22) | 13 (1,23) | -7 (-23,7) | 0.02 |
|  | CS | -3 (-13,9) | 0 (-14,16) | 4 (-11,19) |  |  |
| alpha-hydroxybutyrate | Peripheral | 2 (-3,8) | 9 (-2,15) | 10 (1,18) | 9 (-5,17) | 0.03 |
|  | CS | 3 (-4,7) | 3 (-1,21) | 10 (-4,18) |  |  |
| alpha-ketoglutarate | Peripheral | 4 (-8,11) | 4 (-6,18) | 5 (-4,28) | 15 (-5,30) | 0.33 |
|  | CS | 4 (-7,6) | -2 (-16,18) | 8 (-9,17) |  |  |
| aminoisobutyric acid | Peripheral | 6 (-6,15) | 11 (8,18) | 21 (9,37) | 32 (15,47) | 0.0006 |
|  | CS | 2 (-4,6) | 13 (3,23) | 27 (5,34) |  |  |
| AMP | Peripheral | 12 (-23,62) | 53 (16,120) | 40 (2,237) | 79 (26,186) | 0.004 |
|  | CS | 16 (-37,63) | 16 (4,93) | 24 (2,90) |  |  |
| anthranilic acid | Peripheral | 2 (-15,8) | -3 (-11,12) | -2 (-18,8) | -4 (-21,14) | 0.74 |
|  | CS | -4 (-22,3) | -9 (-17,8) | -12 (-22,12) |  |  |
| arginine | Peripheral | 0 (-6,8) | 2 (-4,6) | 2 (-5,7) | 1 (-12,10) | 0.86 |
|  | CS | -2 (-5,3) | -4 (-9,4) | -2 (-7,5) |  |  |
| asparagine | Peripheral | -1 (-8,3) | -1 (-8,3) | -2 (-10,3) | 4 (-13,11) | 0.56 |
|  | CS | -1 (-8,2) | -1 (-7,3) | -6 (-9,1) |  |  |
| beta-hydroxybutyrate | Peripheral | 26 (11,47) | 87 (13,155) | 49 (16,191) | 38 (-40,134) | 0.0006 |
|  | CS | 52 (32,68) | 44 (14,177) | 39 (14,153) |  |  |
| betaine | Peripheral | 0 (-1,2) | 0 (-1,2) | -1 (-2,2) | 0 (-4,3) | 0.44 |
|  | CS | -1 (-3,1) | -1 (-3,1) | -2 (-3,1.1) |  |  |
| bilirubin | Peripheral | 0 (-7,13) | 15 (-1,29) | 16 (-3,29) | 29 (-15,58) | 0.08 |
|  | CS | -1 (-6,10) | 5 (-6,13) | 8 (-13,19) |  |  |
| cAMP | Peripheral | 9 (-6,31) | -5 (-26,28) | 6 (-25,20) | 11 (-22,43) | 0.48 |
|  | CS | -2 (-24,16) | 3 (-2,10) | 3 (-8,26) |  |  |
| carnitine | Peripheral | -1 (-3,3) | 1 (-3,4) | -3 (-8,5) | -6 (-12,1) | 0.29 |
|  | CS | -1 (-3,1) | -3 (-8,0) | -4 (-11,0) |  |  |
| choline | Peripheral | 0 (-2,6) | -4 (-7,2) | -3 (-6,-2) | 1 (-12,19) | 0.03 |
|  | CS | -1 (-9,2) | -3 (-5,0) | -5 (-7,-3) |  |  |
| cis/trans hydroxyproline | Peripheral | 1 (-11,6) | -5 (-9,3) | -10 (-12,-2) | -10 (-22,12) | 0.12 |
|  | CS | -6 (-12,0) | -10 (-18,-1) | -19 (-24,-10) |  |  |
| citrate | Peripheral | -3 (-5,0) | 0 (-4,5) | -2 (-7,4) | 12 (-2,24) | 0.09 |
|  | CS | -1 (-4,5) | 0 (-3,2) | 0 (-10,2) |  |  |
| citrulline | Peripheral | 2 (-3,5) | -1 (-3,5) | 1 (-7,3) | -8 (-20,-4) | 0.01 |
|  | CS | 0 (-10,1) | -2 (-4,1) | -3 (-10,-1) |  |  |
| CMP | Peripheral | 4 (-16,119) | 17 (3,170) | 17 (-28,77) | 50 (24,113) | 0.12 |
|  | CS | -13 (-40,31) | 21 (-38,66) | -4 (-34,91) |  |  |
| creatine | Peripheral | 1 (-3,5) | 3 (0,11) | 3 (-3,8) | -6 (-21,8) | 0.18 |
|  | CS | -1 (-5,5) | 1 (-3,10) | 4 (-8,9) |  |  |
| creatinine | Peripheral | 0 (-2,2) | -1 (-2,1) | -1 (-1,1) | 2 (-1,7) | 0.16 |
|  | CS | -1 (-5,1) | 0 (-2,1) | -2 (-4,0) |  |  |
| cystathionine | Peripheral | 1 (-8,15) | -5 (-25,16) | -5 (-24,24) | -15 (-27,0) | 0.22 |
|  | CS | 12 (-2,24) | -3 (-17,17) | -7 (-16,6) |  |  |
| cytosine | Peripheral | -5 (-12,20) | -5 (-14,16.4) | -5 (-15,6) | 3 (-12,21) | 0.81 |
|  | CS | -7 (-25,5) | -5 (-15,5) | -3 (-13,6) |  |  |
| dimethylglycine | Peripheral | -1 (-2,3) | -1 (-5,3) | -2.4 (-5,1) | 2 (-8,8) | 0.52 |
|  | CS | -2 (-6,2) | -4 (-8,-1) | -8 (-9,-5) |  |  |
| F16DP/F26DP/G16DP | Peripheral | 3 (-36,40) | 29 (-12,60) | 2.7 (-29,56) | 21 (-38,150) | 0.69 |
|  | CS | -31 (-42,25) | -15 (-37,22) | -7 (-55,42) |  |  |
| F1P/F6P/G1P/G6P | Peripheral | -4 (-13,15) | 0 (-9,18) | 16 (-5,30) | 19 (-12,55) | 0.50 |
|  | CS | -8 (-19,5) | -1 (-15,9) | -6 (-13,2) |  |  |
| fructose/glucose/galactose | Peripheral | 0 (-4,4) | 1 (-3,8) | 0 (-1,4) | -10(-21,-2) | 0.03 |
|  | CS | -2 (-4,1) | -1 (-6,3) | -1 (-6,3) |  |  |
| fumarate/maleate | Peripheral | -11 (-19,36) | 1 (-9,59) | 12 (-6,38) | 4 (-27,49) | 0.13 |
|  | CS | -4 (-16,8) | 0.4 (-19,21) | -5 (-19,13) |  |  |
| GDP | Peripheral | 22 (-27,72) | 63 (32,252) | 55 (7,261) | 69 (21,143) | 0.003 |
|  | CS | 21 (-23,80) | 40 (-9,128) | 31 (-19,220) |  |  |
| gentisate | Peripheral | -1 (-11,6) | -14 (-22,1) | -17 (-30,-5) | -34 (-42,-22) | 0.0006 |
|  | CS | 2 (-13,11) | -16 (-21,-4) | -15 (-28,-8) |  |  |
| glucose | Peripheral | -1 (-3.9,3) | 2 (-7,8) | -1 (-5,10) | -7 (-20,11) | 0.50 |
|  | CS | -4 (-9,-2) | -4 (-7,7) | -6 (-11,3) |  |  |
| glutamate | Peripheral | 0 (-7,15) | 10 (-3,19) | 3 (-5,17) | -35 (-63,11) | 0.001 |
|  | CS | 27 (4,84) | 10 (-7,34) | 1 (-9,52) |  |  |
| glutamine | Peripheral | 1 (-3,4) | 4 (-4,8) | 4 (2,6) | 7 (0,11) | 0.03 |
|  | CS | -2 (-5,1) | 1 (-2,4) | 1 (-3,6) |  |  |
| glycerol | Peripheral | 35 (-5,103) | 48 (9,108) | 16 (2,51) | -5 (-71,19) | 0.006 |
|  | CS | 30 (-14,58) | 43 (-18,87) | -6 (-22,20) |  |  |
| glycine | Peripheral | 0 (-4,5) | -5 (-8,0) | -6 (-12,2) | 1 (-11,9) | 0.13 |
|  | CS | -2 (-8,3) | -1 (-11,-1) | -2 (-9,2) |  |  |
| glycocholate | Peripheral | -6 (-32,15) | -25 (-41,14) | -30 (-54,8) | -51 (-71,-27) | 0.009 |
|  | CS | 0 (-12,10) | -26 (-38,-11) | -24 (-50,12) |  |  |
| glycodeoxycholate/glycochenodeoxcholate | Peripheral | -11 (-27,1) | -40 (-54,-20) | -58 (-69,-3) | -54 (-79,-27) | 0.002 |
|  | CS | -16 (-27,1) | -38 (-55,-21) | -62 (-68,-4) |  |  |
| GMP | Peripheral | 21 (-8,43) | 59 (15,216) | 92 (23,289) | 58 (16,172) | 0.01 |
|  | CS | 5 (-28,57) | 10 (-17,34) | 34 (-4,69) |  |  |
| guanosine | Peripheral | 9 (-5,26) | 2 (-12,11) | 8 (-2,42) | -1 (-16,23) | 0.61 |
|  | CS | 52 (9,87) | 14 (0,53) | 30 (7,52) |  |  |
| histamine | Peripheral | -9 (-30,7) | -13 (-32,31) | -12 (-38,5) | 0.4 (-26,31) | 0.33 |
|  | CS | -2 (-26,12) | -23 (-42,5) | -16 (-44,28) |  |  |
| histidine | Peripheral | -1 (-6,2) | 0.9 (-5,9) | 3 (-1,6) | 1.8 (-11,6) | 0.09 |
|  | CS | -5 (-9,0) | -2 (-7,5) | -1 (-4,0) |  |  |
| homocysteine | Peripheral | 0 (-10,5) | -4 (-14,8) | -1 (-8,2) | -5 (-11,0) | 0.48 |
|  | CS | -3 (-12,6) | 4 (-9,6) | -5 (-10,2) |  |  |
| homogentistate | Peripheral | -6 (-11,7) | -1 (-23,14) | -9 (-23,1) | -13 (-24,8) | 0.29 |
|  | CS | -6 (-20,3) | 0 (-14,9) | -11 (-20,-1) |  |  |
| hydroxyphenylacetate | Peripheral | 8 (-11,12) | -1 (-12,12) | -6 (-18,18) | -18.1 (-33,8) | 0.12 |
|  | CS | -7 (-18,-1) | -15 (-21,1) | -13 (-23,-4) |  |  |
| hyodeoxy/ursodeoxy/chenodeoxy/deoxycholate | Peripheral | -6 (-13,5) | -14 (-28,-1) | -26 (-43,-16) | -48 (-55,-32) | 0.0006 |
|  | CS | -7 (-21,3) | -18 (-23,-10) | -30 (-40,-9) |  |  |
| hypoxanthine | Peripheral | 3 (-12,37) | 10 (-31,25) | -3.6 (-17,22) | -15 (-30,87) | 0.58 |
|  | CS | 16 (-25,63) | -10 (-33,29) | 0 (-12,35) |  |  |
| IMP | Peripheral | 24 (-32,46) | 32 (4,141) | 44 (-18,237) | 50 (-8,172) | 0.03 |
|  | CS | 2 (-33,51) | 46 (-16,98) | 18 (-26,158) |  |  |
| indole 3-propionate | Peripheral | 10 (-14,30) | -12 (-23,23) | -12 (-42.9) | -20 (-57,-9) | 0.007 |
|  | CS | 0 (-26,27) | 0 (-11,18) | -6 (-19,39) |  |  |
| indoxylsulfate | Peripheral | -7 (-15,2) | -12 (-21,0) | -8 (-23,2) | -29 (-36,-1) | 0.04 |
|  | CS | -7 (-13,-1) | -13 (-20,-9) | -13 (-18,-4) |  |  |
| inosine | Peripheral | 8 (-9,27) | -18 (-31,15) | -6 (-32,30) | 7 (-17,40) | 0.04 |
|  | CS | 33 (-15,118) | -10 (-39,56) | -3 (-17,71) |  |  |
| inositol | Peripheral | 1 (-7,8) | -7 (-15,3) | -1 (-14,11) | -6 (-15,1) | 0.28 |
|  | CS | 3 (-8,11) | 3 (-4,7) | -4 (-11,11) |  |  |
| isocitrate | Peripheral | -3 (-11,0) | 1 (-6,6) | 0 (-10,6) | 3 (-6,15) | 0.27 |
|  | CS | -1 (-8,5) | -5 (-11,4) | -7 (-10,4) |  |  |
| isoleucine | Peripheral | -1 (-2,2) | 0 (-8,3) | 4 (-8,9) | 4 (-3,9) | 0.04 |
|  | CS | -2 (-4,2) | -2 (-6,1) | -1 (-7,3) |  |  |
| kynurenic acid | Peripheral | 1 (-3,10) | -2 (-9,13) | -7 (-17,-2) | -19 (-26,3) | 0.03 |
|  | CS | -4 (-12,4) | -13 (-21,-3) | -24 (-32,-11) |  |  |
| kynurenine | Peripheral | -3 (-8,0) | -5 (-20,0) | -3 (-9,6) | -4 (-15,5) | 0.09 |
|  | CS | 0 (-3,2) | -2 (-12,5) | 0 (-9,6) |  |  |
| lactate | Peripheral | 0 (-10,10) | -8 (-23,-2) | -11 (-26,1) | 7 (-6,88) | 0.002 |
|  | CS | 19 (-5,26) | -5 (-22,17) | -8 (-16,15) |  |  |
| lactose | Peripheral | -6 (-17,19) | 14 (-10,62) | 14 (-9,32) | 1 (-9,15) | 0.36 |
|  | CS | -7 (-23,11) | -2 (-8,17) | 12 (-7,24) |  |  |
| leucine | Peripheral | 2 (-3,4) | 0 (-4,5) | 5 (-6,9) | 6 (4,17) | 0.009 |
|  | CS | 0 (-3,4) | -2 (-5,3) | 0 (-3,4) |  |  |
| lithocholate | Peripheral | -8 (-21,10) | -3 (-20,5) | -16 (-24,5) | -30 (-39,-6) | 0.01 |
|  | CS | 7 (-6,23) | 0 (-14,23) | -2 (-31,24) |  |  |
| lysine | Peripheral | -2 (-7,3) | -1 (-8,10) | 1 (-3,6) | 6 (-3,9) | 0.58 |
|  | CS | -3 (-10,0) | -3 (-8,5) | -2 (-11,9) |  |  |
| malate | Peripheral | -3 (-20,18) | -8 (-13,42) | 0 (-14,27) | 42 (-4,66) | 0.17 |
|  | CS | -2 (-7,7) | 6 (-2,27) | 14 (2,24) |  |  |
| methionine | Peripheral | -1 (-4,2) | -1 (-10,3) | -1 (-10,9) | 1 (-11,21) | 0.42 |
|  | CS | -3 (-8,0) | -2 (-8,0) | -3 (-9,-1) |  |  |
| methylmalonate | Peripheral | -6 (-17,10) | -4 (-21,20) | -4 (-13,23) | -9 (-24,3) | 0.32 |
|  | CS | -2 (-14,6) | -4 (-20,2) | -2 (-12,3) |  |  |
| N-carbomoyl-beta-alanine | Peripheral | 5 (-2,7) | 1 (-6,10) | 7 (-2,15) | 8 (4,18) | 0.04 |
|  | CS | -4 (-12,5) | -6 (-8,0) | -6 (-9,7) |  |  |
| niacinamide | Peripheral | -1 (-15,2) | 5 (-11,23) | 1 (-13,28) | 50 (-19,179) | 0.74 |
|  | CS | -11 (-19,11) | 1 (-22,25) | -5 (-11,13) |  |  |
| NMMA | Peripheral | 1 (-5,5) | 7 (-2,11) | 5 (2,12) | 8 (-5,23) | 0.06 |
|  | CS | -5 (-11,2) | -1 (-4,6) | 3 (-9,12) |  |  |
| ornithine | Peripheral | 1 (-7,7) | 0 (-5,9) | 2 (-4,9) | -11 (-25,5) | 0.20 |
|  | CS | -5 (-12,3) | -4 (-9,11) | 6 (-3,14) |  |  |
| oxalate | Peripheral | -15 (-23,32) | -10 (-30,3) | -4 (-30,12) | -31 (-48,21) | 0.12 |
|  | CS | -4 (-16,20) | -16 (-32,-2) | -7 (-38,28) |  |  |
| pantothenate | Peripheral | -3 (-11,2) | -2 (-8,2) | 2 (-6,6) | -12 (-23,3) | 0.36 |
|  | CS | 2 (-4,6) | -1 (-13,11) | 4 (-9,14) |  |  |
| PEP | Peripheral | 0 (-12,25) | -14 (-22,7) | -8 (-26,13) | -14 (-39,27) | 0.23 |
|  | CS | -2 (-25,27) | -8 (-39,17) | -9 (-33,27) |  |  |
| phenylalanine | Peripheral | -1 (-4,3) | -1 (-6,2) | -1 (-8,4) | 1 (-3,7) | 0.45 |
|  | CS | -2 (-11,0) | -3 (-6,0) | -3 (-7,-1) |  |  |
| phosphocholine | Peripheral | -1 (-4,4) | -3 (-6,4) | -2 (-5,3) | 1 (-8,6) | 0.81 |
|  | CS | -2 (-6,1) | -2 (-5,0) | -1 (-5,1) |  |  |
| phosphocreatine | Peripheral | -1 (-18,12) | 9 (-5,18) | 7 (-9,37) | 62 (26,87) | 0.002 |
|  | CS | 6 (-2,10) | -3 (-10,11) | -6 (-16,22) |  |  |
| phosphoglycerate | Peripheral | -3 (-23,17) | 2 (-27,36) | -17 (-30,17) | -12 (-26,58) | 0.86 |
|  | CS | -22 (-39,6) | -6 (-29,10) | -11 (-35,26) |  |  |
| proline | Peripheral | -1 (-2,1) | -1 (-5,1) | -3 (-5,0) | -4 (-10,1) | 0.04 |
|  | CS | -3 (-4,1) | -3 (-7,0) | -5 (-8,-2) |  |  |
| pyruvate | Peripheral | -5 (-14,7) | 1 (-11,30) | 32 (-15,48) | 67 (17,136) | 0.002 |
|  | CS | 26 (-6,50) | 31 (-1,81) | 71 (2,129) |  |  |
| quinolinate | Peripheral | -4 (-17,4) | -2 (-19,15) | -4 (-18,38) | -7 (-33,14) | 0.50 |
|  | CS | -7 (-14,4) | -16 (-25,-1) | -7 (-15,4) |  |  |
| ribose 5-P/ribulose 5-P | Peripheral | -14 (-30,36) | -8 (-34,30) | 12 (-25,28) | 23 (-14,98) | 0.68 |
|  | CS | -23 (-43,24) | 10 (-38,37) | -2 (-54,41) |  |  |
| SDMA | Peripheral | -5 (-10,1) | -4 (-16,13) | 1 (-5,13) | 3 (-6,15) | 0.12 |
|  | CS | -5 (-14,9) | -2 (-17,14) | -3 (-10,4) |  |  |
| sebacate | Peripheral | 2 (-8,5) | 1 (-4,14) | 4 (-5,20) | 4 (-4,11) | 0.41 |
|  | CS | -3 (-7,1) | -1 (-7,5) | -6 (-8,-1) |  |  |
| serine | Peripheral | 1 (-4,5) | 3 (-3,12) | 6 (-3,11) | -6 (-17,-2) | 0.04 |
|  | CS | -2 (-6,5) | 0 (-5,12) | 1 (-1,14) |  |  |
| serotonin | Peripheral | 37 (-15,57) | 63 (11,154) | 57 (3,381) | 57 (-3,92) | 0.002 |
|  | CS | 11 (-14,42) | 64 (-3,108) | 16 (-9,117) |  |  |
| sorbitol | Peripheral | -1 (-4,3) | -4 (-10,1) | -4 (-13,2) | -26 (-43,-5) | 0.0006 |
|  | CS | -1 (-3,1) | -4 (-7,-2) | -7 (-11,-4) |  |  |
| spermidine | Peripheral | -5 (-16,14) | -10 (-23,31) | -5 (-21,31) | 17 (-11,72) | 0.65 |
|  | CS | -11 (-28,1) | -7 (-22,23) | 3 (-20,34) |  |  |
| suberate | Peripheral | -8 (-20,7) | -3 (-20,13) | -5 (-15,4) | -4 (-19,13) | 0.28 |
|  | CS | -7 (-15,-2) | -11 (-15,-3) | -6 (-17,5) |  |  |
| succinate | Peripheral | 3 (-18,27) | -4 (-29,63) | 8 (-16,52) | 29 (6,69) | 0.15 |
|  | CS | 6 (-17,52) | 9 (-3,22) | 13 (-9,30) |  |  |
| sucrose | Peripheral | -11 (-28,11) | -17 (-33,20) | -18 (-34.-2) | -39 (-52,-8) | 0.01 |
|  | CS | -13 (-24,43) | -16 (-27,1) | -15 (-30,50) |  |  |
| taurine | Peripheral | 3 (-7,17) | 12 (2,50) | 13 (5,49) | 8 (-3,24) | 0.007 |
|  | CS | 4 (-3,14) | 15 (-5,29) | 14 (0,28) |  |  |
| taurocholate | Peripheral | -3 (-29,22) | -39 (-55,7) | -29 (-61,4) | -54 (-82,-9) | 0.02 |
|  | CS | 0 (-18,11) | -8(-35,0) | -26 (-61,25) |  |  |
| taurodeoxycholate/taurochenodeoxycholate | Peripheral | -9 (-31,2) | -46 (-59,-26) | -61 (-75,12) | -63 (-81,-20) | 0.009 |
|  | CS | -14 (-33,7) | -43 (-59,-8) | -64 (-78,15) |  |  |
| thiamine | Peripheral | -1 (-10,9) | 0 (-5,14) | 7 (-6,15) | -17 (-34,-1) | 0.04 |
|  | CS | -10 (-15,-1) | -3.4 (-9,4) | -3 (-9,3) |  |  |
| threonine | Peripheral | -1 (-4,4) | -1 (-5,1) | -1 (-5,1) | 2 (-18,5) | 0.63 |
|  | CS | -4 (-8,1) | -5 (-7,2) | -5 (-7,3) |  |  |
| thyroxine | Peripheral | 2 (-3,5) | 0 (-6,2) | -1 (-5,2) | -60 (-72,-34) | 0.002 |
|  | CS | -4 (-9,3) | -5 (-8,2) | -6 (-10,0) |  |  |
| triiodothyronine | Peripheral | -7 (-17,9) | -1 (-9,24) | -1 (-13,17) | -39 (-51,-18) | 0.005 |
|  | CS | -1 (-24,16) | -1 (-19,21) | 2 (-14,23) |  |  |
| trimethylamine-N-oxide | Peripheral | -1 (-3,5) | -2 (-8,8) | -3 (-12,6) | -16 (-28,6) | 0.07 |
|  | CS | -1 (-5,3) | 3 (-5,7) | 2 (-11,7) |  |  |
| tryptophan | Peripheral | -6 (-13,1) | -9 (-14,-4) | -9 (-17,2) | -9 (-19,3) | 0.06 |
|  | CS | -7 (-13,-3) | -7 (-13,-4) | -6 (-14,-2) |  |  |
| tyrosine | Peripheral | 0 (-3,4) | -3 (-9,1) | -3 (-10,2) | -3 (-11,1) | 0.09 |
|  | CS | -3 (-12,-1) | -6 (-10,0) | -7 (-13,-3) |  |  |
| UDP-galactose/UDP-glucose | Peripheral | -6 (-31,81) | 49 (-6,75) | 19 (-9,66) | 58 (1,100) | 0.03 |
|  | CS | -4 (-38,23) | 38 (-11,70) | 21 (0,81) |  |  |
| urate | Peripheral | 0 (-1,2) | -1 (-3,6) | 3 (-2,10) | 2 (-2,11) | 0.64 |
|  | CS | -1 (-3.,2) | -3 (-6,4) | 0 (-3,1) |  |  |
| uridine | Peripheral | 3 (-1,10) | 3 (-3,8) | 3 (-1,11) | -40 (-52,-27) | 0.0006 |
|  | CS | 7 (0,13) | -3 (-6,3) | 0 (-3,7) |  |  |
| valine | Peripheral | 0 (-3,2) | -2 (-4,3) | -1 (-3,4) | 1 (-4,3) | 0.64 |
|  | CS | -1 (-4,0) | -2 (-5,0) | -3 (-5,1) |  |  |
| xanthine | Peripheral | 6 (-6,20) | 10 (-2,29) | 17 (5,35) | 61 (16,81) | 0.0006 |
|  | CS | 6 (-8,22) | 16 (-4,21) | 20 (-0,40) |  |  |
| xanthosine | Peripheral | -7 (-60,7) | -6 (-44,43) | 4 (-37,40) | 3 (-31,47) | 0.04 |
|  | CS | -5 (-36,51) | -6 (-24,60) | -4 (-21,229) |  |  |
| xanthurenate | Peripheral | 4 (-9,9) | -11 (-21,-3) | -12 (-32,7) | -29 (-39,-14) | 0.003 |
|  | CS | -4 (-12,2) | -7(-29,0) | -19 (-34,2) |  |  |

Table S2: Mean changes from baseline observed in the control cath population. Even without FDR-correction, only three of the total number of significantly changed metabolites from the pacing cohort demonstrated directionally similar changes in the control group.

|  | Change after cath | p-value |
| --- | --- | --- |
| **Amino Acid Metabolism** |  |  |
| 2-aminoadipate | 9.1% | NS |
| α-hydroxybutyrate | 4.0% | NS |
| gentisate | 19.5% | NS |
| glutamate | 33.0% | NS |
| **Tryptophan Hydrolase Metabolites** |  |  |
| 3-hydroxyanthranilic acid | -12.4% | 0.009 |
| 5-hydroxytryptophan | -0.7% | NS |
| indole 3-propionate | 3.7% | NS |
| indoxylsulfate | -1.9% | NS |
| kynurenic acid | 7.9% | NS |
| serotonin | 4.7% | NS |
| xanthurenate | -2.5% | NS |
| **Purine Metabolism** |  |  |
| adenosine | -27.2% | 0.048 |
| ADP | -27.0% | NS |
| allantoin | 0.3% | NS |
| AMP | -2.6% | NS |
| GDP | -18.4% | NS |
| GMP | -23.5% | NS |
| IMP | N/A | N/A |
| inosine | -13.6% | NS |
| xanthine | 1.8% | NS |
| **Pyrimidine Metabolism** |  |  |
| aminoisobutyric acid | 19.3% | NS |
| uridine | -1.9% | NS |
| **Glycolysis/Carbohydrate Metabolism** |  |  |
| fructose/glucose/galactose | -2.7% | NS |
| lactate | -5.1% | NS |
| pyruvate | -14.4% | 0.01 |
| sorbitol | -4.4% | NS |
| sucrose | -3.1% | NS |
| UDP-galactose/UDP-glucose | -25.0% | 0.04 |
| **Bile Acid Metabolism** |  |  |
| glycocholate | -15.8% | NS |
| glycodeoxycholate/ glycochenodeoxycholate | 1.4% | NS |
| hyodeoxy/ursodeoxy/ chenodeoxy/deoxycholate | -25.4% | 0.012 |
| lithocholate |  |  |
| taurine | -0.9% | NS |
| taurocholate | -17.0% | NS |
| taurodeoxycholate/ taurochenodeoxycholate | -8.9% | NS |
| **Lipolysis** |  |  |
| β-hydroxybutyrate | 6.8% | NS |
| glycerol | 20.5% | NS |
| **Thyroid Hormones** |  |  |
| thyroxine | -10.5% | NS |
| triiodothyronine | N/A | N/A |
| **Other Metabolic Pathways** |  |  |
| aconitate | 14.7% | NS |
| α-glycerophosphocholine | -4.6% | NS |
| phosphocreatine | 39.1% | NS |
| thiamine | 8.3% | NS |
